# Nanoscale devices for linkerless long-term single-molecule observation

**DOI:** 10.1016/j.copbio.2016.02.013

**Published:** 2016-03-15

**Authors:** Niccolò Banterle, Edward A Lemke

**Affiliations:** Structural and Computational Biology Unit and Cell Biology and Biophysics Unit, European Molecular Biology Laboratory (EMBL), Meyerhofstrasse 1, 69117 Heidelberg, Germany

## Abstract

Total internal reflection fluorescence microscopy (TIRFM) can offer favorably high signal-to-noise observation of biological mechanisms. TIRFM can be used routinely to observe even single fluorescent molecules for a long duration (several seconds) at millisecond time resolution. However, to keep the investigated sample in the evanescent field, chemical surface immobilization techniques typically need to be implemented. In this review, we describe some of the recently developed novel nanodevices that overcome this limitation enabling long-term observation of free single molecules and outline their biological applications. The working concept of many devices is compatible with high-throughput strategies, which will further help to establish unbiased single molecule observation as a routine tool in biology to study the molecular underpinnings of even the most complex biological mechanisms.

## Introduction

Observation of molecular properties at the single-molecule (SM) level has proven to be an extremely powerful tool for the study of molecular mechanisms. By direct visualization of one molecule at a time it is possible to measure distributions of molecular states and identify possible functional subpopulations as well as transient states that are otherwise unidentifiable from the bulk ensemble average [1,2].

There are several aspects, which need to be considered when designing a SM experiment. One essential consideration is to optimize the spatial and temporal scales relevant for the process under observation. Conventional SM microscopy, being based on localization precision, can provide spatial information in the tens of nanometer regime and hence on the diffusion properties of the observed sample, while fluorescence resonance energy transfer (FRET) studies can be used to monitor distances between up to 10 nm enabling structural conformation monitoring. In both cases (although with different dependences [3,4]) the achievable resolution will depend upon the signal to noise ratio (SNR). In fluorescent based systems, the signal is defined by the limited number of photons emitted by a single fluorophore (typically of the order of 10^3^–10^6^ [5]) and by the efficiency of the detection system. One strategy to increase the SNR is to increase the signal; this can be accomplished with an increase in the exposure/binning time, which will lead to a decrease in the temporal resolution, or increasing the excitation power, leading to an overall reduced observation time before the occurrence of dye bleaching. The other way is to reduce noise, which is mainly contributed by out of focus molecules, autofluorescence of the medium/cover-slip and electronic noise of the detector, by employing the most natural solution of introducing some form of optical sectioning. Given this consideration, several microscopy techniques that provide high optical sectioning have been implemented to observe the fluorescence emission from individual molecules with high signal-to-noise ratios.

**Figure 1:**
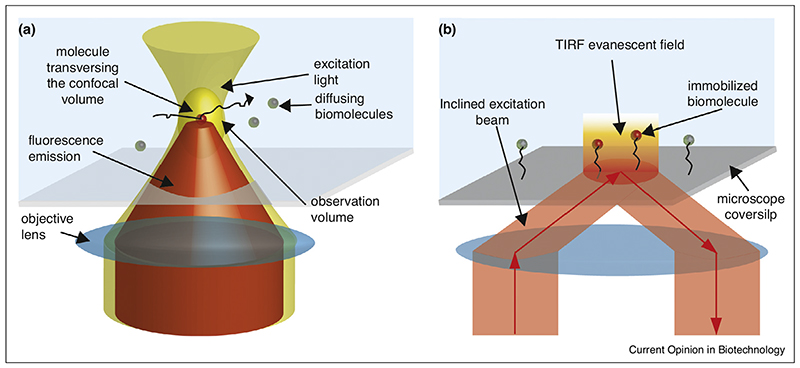
Detection schemes typically adopted for single-molecule observation are confocal microscopy **(a)** and TIRFM **(b)**. In confocal spectroscopy single molecules (spheres) are excited from a focalized laser (depicted in yellow in panel a) and the fluorescence signal (depicted in red, in panel a) is emitted by molecule when transiently crossing the confocal volume. In TIRFM an inclined laser (colored in red in panel b, with arrow indicating the direction of light propagation) at the critical angle is used to generate an evanescent excitation field used to excite molecules attached to the surface of the microscopic glass.

The most commonly established single-molecule fluorescent techniques are confocal spectroscopy and total internal reflection fluorescence microscopy (TIRFM) [6]. Confocal spectroscopy allows the observation of single molecules in solution while they diffuse through the confocal excitation volume. In brief, the fluorescent sample is observed in solution at a concentration (~100 picomolar) such that maximal one molecule is present in the diffraction-limited volume generated by focusing a laser beam with a high numerical aperture (NA) objective (Figure 1a). The fluorescence signal is then collected through the same objective and recorded after optical sectioning through a pinhole onto a single photon sensitive point detector. Single molecules diffusing through the confocal volume appear in the recorded signal as a transient higher-signal burst [7]. The main drawback of this technique is that the observation time of a single molecule is limited by the diffusion time of the molecule out of the confocal volume (~ of the order of a few milliseconds).

To observe molecular processes longer, the commonly adopted approach is to chemically immobilize the fluorescent molecules (e.g. by using the strong binding between biotin and streptavidin) on a surface (e.g. a coverslip) and then visualize it by either confocal microscopy [8] or, more commonly, by TIRFM [9], in which an expanded and inclined laser beam is incident on the microscope coverslip to water interface at the critical angle to generate an evanescent excitation field that rapidly decays within a few hundred nanometers (Figure 1b). However, depending on the molecule of interest, finding the appropriate immobilization strategy can be a challenge. In particular, proteins are not easy to site-specifically functionalize in many different ways. In fact, already installing site-specific appropriate pairs of dyes for FRET studies, which are commonly used to study structure and dynamics of molecular machines, is frequently a major hurdle [10–12]. Adding another modification for immobilization site is, for many proteins, thus out of reach. In addition, immobilization can alter the molecular dynamics of the sample and even impede biological function, but appropriate choice of the immobilization site and proper surface passivation can overcome these concerns in many cases [9].

For these reasons, several techniques have been developed in recent years to observe fluorescently labeled molecules in solution, at the single-molecule level, for long times without the need for a covalent linkage. One elegant and effective way is to encapsulate molecules into nano-containers, such as polymer cages, lipid droplets, which then in turn can be immobilized at the surface. In particular, liquid droplets have been widely used in cases where the biomolecule of interest was compatible with the droplet formation procedure. Although these techniques are elegant and powerful, they are conceptually different than the devices presented in this review and the interested reader is referred to other literature on these procedures [9,13–18].

In this review, we outline recently developed strategies that have been adopted to increase the observation time of fluorescently labeled biomolecules, without the need of covalent chemical immobilization, using nanodevices, and their recent implementation (Table 1).

### Active compensation of Brownian motion

**Figure 2:**
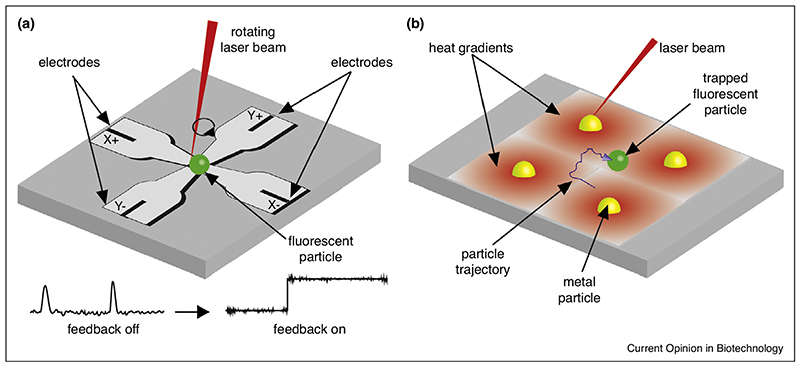
In the ABEL trap **(a)** the fluorescence emitted from a molecule excited with a rapidly rotating beam is used to monitor its position, which is used in a closed-feedback mode to generate the voltage at four electrodes (X+, X−, Y+, Y−). The generated electric field counteracts Brownian diffusion, keeping the particle in position. A similar concept is applied in a thermophoretic trap **(b)** in which the laser beam excites plasmons of metallic nanoparticles, generating thermal gradients (red gradients) which trap the single molecule.

An elegant strategy developed to increase the observation time of fluorescent nanoscale particles/objects in solution is the anti-Brownian electrokinetic (ABEL) trap [19]. The concept of the device relies on two steps. In the first, the diffusing behavior of the observed particle is monitored by fluorescence microscopy. Secondly, the measured displacement is used by a feedback loop system to apply a voltage such that the electrokinetic force resulting from the generated field exactly balances the diffusion process, keeping the particle in a stable position. To achieve this, the ABEL trap consists of four microelectrodes arranged in a diamond shape in a microfluidic cell (Figure 2a). In the first ABEL trap [20], the microfluidic cell was made of polydimethylsiloxane (PDMS), the position of the molecule was monitored by fluorescence imaging, and the displacement quantification as well as the feedback signal generation was software driven. In this configuration, the size of the smallest object that could be trapped was set by the finite response time of the feedback loop, and allowed the trapping of 20 nm polystyrene nanospheres. In a successive development [21] the substitution of PDMS with a microfluidic chamber built entirely out of glass allowed the first trapping of biological objects, such as the tobacco mosaic virus (300 × 15 nm) and lipid vesicles (100 nm diameter). In the next generation of the device [22,23], to reduce the size of the trapped object down to the single-molecule level, the software feedback loop was substituted for an active hardware-based closed loop. In this configuration, the position of the trapped object was sensed by scanning the fluorescent label with a quickly rotating (40 kHz) laser beam ([24] as developed by Mabuchi [25] and Gratton [26]). With this configuration, in which the microfluidic chamber was made of fused silica to reduce the fluorescence background, it was possible to trap individual molecules (GroEL) and even single fluorophores (Cy3) in aqueous solution. Further developments in the position-estimation algorithm and scanning patterns, as well as novel analysis methods, allowed the conformational modes [27] and kinetics [28^••^] of DNA molecules to be revealed. The combination with multiparameter fluorescence detection allowed monitoring of the conformational dynamics [29] and the effect of oligomerization [30^•^] of light-harvesting pigment protein complexes. The latest generation of the ABEL trap has also been successfully implemented in the study of multi-subunit enzymes, for example, its use revealed the effect of cooperativity in the mammalian group II chaperonin TRiC/CCT [19] and the regulatory mechanism of the F1-ATPase by single-molecule FRET [31^•^]. Meanwhile also ABEL traps were presented that permit three-dimensional trapping [32,33].

Recently, an alternative method has been proposed [34^••^] to trap single molecules in a similar way to the ABEL trap, but which instead of using electrokinetic forces, exploits induced thermophoretic drifts to counteract the singlemolecule Brownian motion. The underlying principle is that if exposed to thermal gradients, single molecules feel an effective potential energy that is proportional to the temperature gradient itself, and the shape of the temperature profile determines that of the potential energy (Figure 2b). By exciting the plasmons on metallic colloids it is possible to generate high local temperature gradients. In utilizing this principle, the trapping of single colloids in a stationary temperature field has been demonstrated [35]. By introducing an active feedback on the laser used to stimulate the plasmon excitation, Braun *et al*. were able to trap l-DNA molecules for more than 500s [34^••^]. The technique is still at the proof of concept level and has not been demonstrated for single molecules, however it allows controlling the motion of molecular object beyond simple trapping, a task that would be non-trivial to perform with the ABEL trap.

### Confinement of the Brownian motion *via* CLIC, CLINT and/or capillaries

Despite its effectiveness for increasing the single-molecule observation time, the design of the ABEL trap is limited to sequential observation of single molecules, because just one molecule at a time can be trapped. Moreover, its manufacture and operation requires advanced technical proficiency. A strategy to achieve free single-molecule observation for a prolonged time with high throughput is to confine the motion of a single molecule along a single imaging plane.

**Figure 3:**
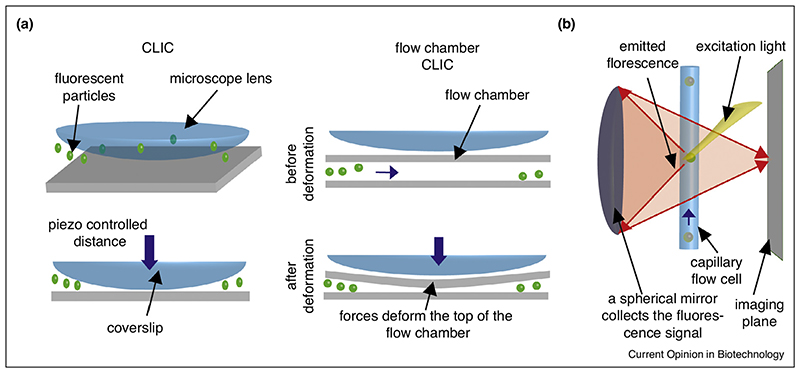
In CLIC **(a)**, a microscopy lens is placed in close vicinity to the coverslip to restrict the molecules’ motion to a 2D thin layer. In the flow chamber CLIC the lens is used to deform the top layer of a flow chamber. **(b)** To achieve high-throughput, single molecules can be imaged using a spherical mirror while flowing through a capillary cell.

A simple method to achieve this goal, a technique known as convex lens-induced confinement (CLIC), has been developed by the Cohen laboratory [36]. In this method, a simple lens is utilized as a confining device (Figure 3a). By placing a plano-convex microscopy lens in close vicinity to the microscope coverslip, a wedge-shaped gap is created which can be tuned to measure a few nanometers in height. The device was able to image freely diffusing lipid vesicles for an extended time (four orders of magnitude longer than for freely diffusing vesicles). In CLIC, molecules stochastically diffuse in and out of the gap, which limits their throughput. To overcome this, CLIC has been implemented in flow-based devices to confine the motion of molecules in 1D channels [37^••^]. In this implementation the spatial confinement is obtained pressing the imaging lens on the flow chamber deforming it bringing the two surfaces constituting it in contact. In such a configuration the separation layer in which the sample is confined can be reduced to 60 nm, providing an increase of up to five fold in signal to noise ratio and at the same time facilitating the sample replacement. In a recent study the CLIC concept has been combined with nano-surface templates in a method named CLINT [38]. In this declination of the CLIC technique the compression is performed on a nano-lithography printed substrate in order to facilitate the loading of molecules into features (e.g. nanopits or nano-channels) [38].

The Takahashi laboratory have established a system to image single molecules flowing through a fused silica capillary cell [39]. A laser beam is focused along the flow cell and the fluorescence emission is collected with a spherical mirror, corrected with an ad hoc lens system and imaged (Figure 3b). Long observation (of the order of seconds) of Cyt c from *Pseudomonas aeruginosa* could be achieved by adopting a flow-and-stop protocol. Despite this significant advancement the method requires a specifically designed imaging setup and the NA of the imaging system (0.7) is quite low, which limits sensitivity of the technique.

### Exploring the high SNR of TIRFM using SWIFT

**Figure 4:**
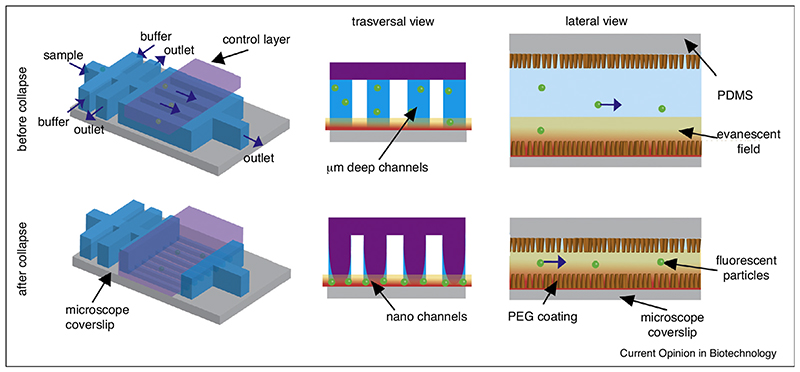
In SWIFT, nanometer-deep channels are reversibly created by collapse through a gas-pressurized pillow-like layer separated from the flow channels by a thin PDMS layer (shown in violet). Different inlets for the buffers and for the sample allow to quickly adjust the concentration, or perform out of equilibrium studies due to the low dead time of the mixer.

As TIRFM of surface immobilized molecules is arguably one of the most widely used methods to study single molecules over a long period, combining TIRFM with single-molecule confinement in nanochannels seems an ideal strategy. Molecules would flow along the surface and could easily be followed using imaging-based technology while being immersed in the thin (~200 nm thick) evanescent excitation field at the surface–water interface that is generated in the TIRFM microscope. However, nanofabrication techniques are typically expensive and nanodevices can be difficult to operate as higher driving pressures are required to overcome the high resistance of the nanochannels [40]. To accomplish an unattached single-molecule observation for a prolonged time with a high SNR and minimal technological requirements, our laboratory recently adapted a technique to generate nanochannels in a PDMS-based microfluidic device [41^••^]. The technique, named single-molecule without immobilization for TIRFM (SWIFT), is based on a multilayer PDMS device in which an upper layer is pressurized to reversibly collapse the underlying microchannels (which are easy to fabricate) with the consequent creation of two nanochannels from each microchannel (Figure 4). Single-molecules can then be observed by TIRFM and tracked while flowing through the channels without leaving the evanescent field. As the technique is based on camera imaging (in fact, it can be operated on any regular TIRF microscope and does not require specialized equipment), several nanochannels can be observed at the same time, allowing for high-throughput studies (several order of magnitude higher throughput than confocal observation, was demonstrated). Before their collapse, the channels are 1 μm high, and the internal surface of the channels can easily be flushed to passivate the device, for example, with polyethylenglycol (PEG), prior to the experiment, yielding the same passivated surface that researchers are used to when using chemical immobilization strategies [9]. Moreover, the reversibility of the collapse can be exploited to re-convert the nanochannels into microchannels to unclog the device in case of temporary obstruction of the nanochannels by, for example, air bubbles or large particles — a common hindrance in nanofluidic devices. The gas permeability of the PDMS was exploited in this device by utilizing nitrogen as a pressurizing gas, enabling oxygen removal from the sample and thus significantly reducing bleaching (frequently more than 50 fold) [42]. In fact, in many cases single dyes did not bleach while traversing along the field of view, so that camera chip size became limiting for how long a molecule could be observed.

Furthermore, SWIFT is based on standard PDMS manufacturing, and therefore a plethora of already designed microfluidic tools are available to be easily implemented [43]. For example, in the current design, a previously developed ultrafast microfluidic mixer was introduced in order to tune the observed sample concentration and possibility to perform non-equilibrium studies with low dead time of 400 ms [44]. In this device the minimum size of the observable sample is limited by the camera exposure rate which needs to be fast enough for the sample not to diffuse substantially during the exposure time, which would otherwise lead to spreading of the fluorescence signal over more pixels. The SWIFT device has been demonstrated to be effective in measuring the dynamics of fluorescently labeled molecules by single-molecule FRET, which allows monitoring of conformational changes and, being a ratiometric technique, it is intrinsically rather independent of illumination inhomogeneity. Holliday junction dynamics, as well as proteins such as the human transglutaminase 2 were monitored for ~ seconds. Also, nucleosomes labeled with a dye pair for FRET were imaged, demonstrating the suitability of the technique for the study of macromolecular complexes. As the molecules are permitted to freely diffuse along the channel, SWIFT observation is easier with larger biomolecules, and is thus perfectly complementary with surface immobilization techniques, for which the increasing size of a protein often creates additional complications involved with installing dyes and surface tethers.

### Confinement of motion in a restricted volume

**Figure 5:**
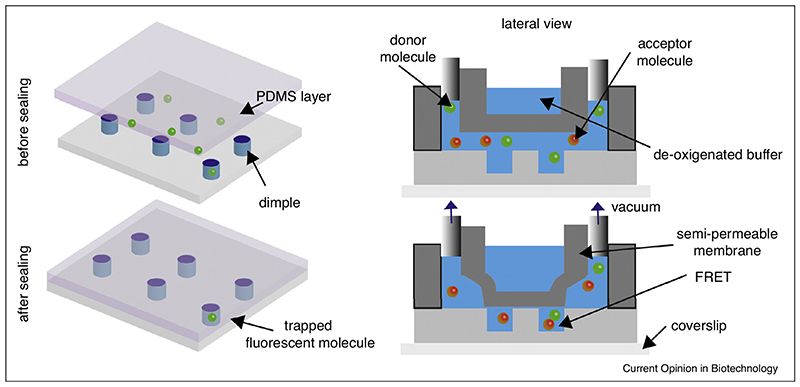
In the dimple device, single molecules randomly enter nano-dimples in which they can be trapped by sealing them under a PDMS layer, allowing single molecule studies at high concentration due to the reduced volume of the dimples.

The last strategy under review is based on confining single molecules in a restricted volume. One way to achieve this task, developed by the Cohen laboratory, is to trap individual molecules in an array of nanofabricated holes or ‘dimples’ measuring 70 nm to 1.3 μm in diameter and 200 nm in depth [45]. The dimples are nanofabricated on a fused silica coverslip and the trapping is accomplished by means of a pneumatically controlled PDMS lid, which can reversibly seal the dimples (Figure 5). As several dimples can be imaged at the same time and the sealing is fully automated, successive cycles of trapping–measuring–refreshing can be performed. The current design of the dimple machine is not compatible with TIRF-type illumination, which limits the signal-to-noise that can be achieved. However the dimple machine is particularly suited for studies in which nano-confinement plays an important role, mimicking the micro-environment of vesicles or some eukaryotic organelles, as demonstrated by the pilot study on ssDNA hybridization [46].

## Conclusions

The need to observe molecular mechanisms at the single-molecule level to capture transient or low-abundance conformational states is clear. However, until the last decade, the observation of fluorescently labeled single molecules was limited to durations of a few milliseconds or involved chemical immobilization of the sample, which is frequently not possible. This greatly limits the number of systems that can be studied in detail. Even after the first demonstration of long-term single-molecule observation these experiments were restricted to laboratories with highly specific technical competence. Only in the past few years the development of novel techniques, here reviewed, enabled the investigation of single-molecule trajectories and molecular reactions for prolonged time in an accessible and cost-effective manner, and in turn, of novel biological mechanisms. These immobilization free methods are synerigistic to elegant developments that permit to enhance the SNR when visualizing single fluorescent molecules, such as the use of nanoantennas or zeromode waveguides (for a review see: [47]). We believe that especially the use of cost-efficient PDMS-based platforms will become widespread, as they are easy to fabricate and can be adapted to suit a variety of applications.

## Figures and Tables

**Table 1:** Summary of the most relevant quantities for the reviewed techniques. In light of their different properties it will become apparent that the methods are rather complementary, and have different pros and cons, such that the ultimate choice depends on the specific application planned. In addition to the SNR and the residence time in the observation volume there are other factors, which are important to consider when deciding the optimal technique for a given experiment, such as concentration and to what extend observation conditions are influencing the physiological behavior of the observed molecule, e.g. due to potential unspecific interaction with device surfaces.

Technique	Max. observation time	Molecules observed simultaneously	Throughput	SNR	Parameters established for	Ref.	Used for e.g. also for
Confocal	~5 ms	1	9000	30	2400 bp dsDNA	[41^••^]	Generally applicable
ABEL	~15s	1	1800 [22]	3.6	10 nt ssDNA	[28^••^]	GroEl, Cy3, TRiC/CCT, F2-ATPase
Termo-phoretic trap	~500 s	1	NA	NA	λ-DNA	[34^••^]	λ-DNA, polystyrene nanoparticles
CLIC	~25 s	100	14,400	10-60	Lipid vesicles	[36]	Myosin
Capillary cell	~6 s	NA	NA	2.9	Cytochrome c	[39]	Cytochrome c
SWIFT	~20 s	30	120,000	12.5 red, 9.3 green	2400 bp dsDNA	[41^••^]	Holliday junction, Transglutaminase 2, Nucleosomes
Dimple	~60 min	200	2000	10 red, 4.6 green	30 nt ssDNA	[45]	ssDNA
